# The Role of Self-Sacrifice in Moral Dilemmas

**DOI:** 10.1371/journal.pone.0127409

**Published:** 2015-06-15

**Authors:** Sonya Sachdeva, Rumen Iliev, Hamed Ekhtiari, Morteza Dehghani

**Affiliations:** 1 Social Science Research Unit, US Forest Service, Evanston, IL, United States of America; 2 Ford School of Public Policy, University of Michigan, Ann Arbor, MI, United States of America; 3 Research Center for Molecular and Cellular Imaging, Iranian National Center for Addiction Studies, Tehran University of Medical Sciences, Tehran, Iran; 4 Translational Neuroscience Program, Institute for Cognitive Science Studies, Tehran, Iran; 5 Department of Psychology, University of Southern California, Los Angeles, CA, United States of America; 6 Brain and Creativity Institute, University of Southern California, Los Angeles, CA, United States of America; Centre national de la recherche scientifique, FRANCE

## Abstract

Centuries’ worth of cultural stories suggest that self-sacrifice may be a cornerstone of our moral concepts, yet this notion is largely absent from recent theories in moral psychology. For instance, in the footbridge version of the well-known trolley car problem the only way to save five people from a runaway trolley is to push a single man on the tracks. It is explicitly specified that the bystander cannot sacrifice himself because his weight is insufficient to stop the trolley. But imagine if this were not the case. Would people rather sacrifice themselves than push another? In Study 1, we find that people approve of self-sacrifice more than directly harming another person to achieve the same outcome. In Studies 2 and 3, we demonstrate that the effect is not broadly about sensitivity to self-cost, instead there is something unique about sacrificing the self. Important theoretical implications about agent-relativity and the role of causality in moral judgments are discussed.

## Introduction

Is the “self” relevant for the moral domain? Though this question might seem trivial at first, it has been a topic of debate within moral philosophy for several decades [1–5]. In particular, philosophers have highlighted a self-other asymmetry when it comes to morally relevant decision-making where actions permissible for the self are not necessarily permissible for others. However, in the recent upsurge in psychological work on moral dilemmas, this discussion has been largely absent. Most studies have been agnostic to the role of the self in morally-motivated decisions–typically, not making a distinction between consequences for the self and others (a notable exception is the relatively new body of work on moral licensing, which suggests that people’s self-perceptions and evaluations, i.e. as moral or immoral individuals, affects their moral behavior on subsequent occasions). In the current work, however, we suggest that the self has a central role to play in the moral domain, and understanding this role is a prerequisite for our understanding of human morality.

One intuitive answer to the question about the role of the self in the moral domain is that acting morally and acting to benefit oneself are inherently contradictory, i.e. moral actions are those that do not promote self-interest. Haidt [6], for example, starts his influential paper by stating, “people are selfish, yet morally motivated”, implying a conflict between morality and self-interest. While Haidt's theory is rather nuanced, a central point he makes is that morality can be seen as a mechanism that controls self-interest and thus make sociality and cooperation possible. When a situation is seen as being a part of the moral realm, the choice which conflicts with selfishness can often be seen as morally right. One illustration is that most people find it harder to stick a pin in the palm of unknown child, than in their own palm suggesting that when in the moral mindset, inflicting pain unto others might be less preferable than inflicting pain unto oneself.

The contrast between acting morally or in one’s self-interest can be found in many aspects of cultural beliefs. For example, self-sacrificial acts are often at the core of many myths and religious teachings [7]. Self-sacrifice is also associated with heroic acts in stories related to national identity [8], and it is often taught as a virtue in children stories [9]. Self-sacrifice for a specific cause can also be a particular powerful signal for the strength of a moral position [10,11]. The recent wave of revolution that spread through the Arab world, known as the Arab Spring, was prompted in a large part by a self-sacrificial act by a fruit vendor in a small village in a distant region of Tunisia. The symbolism of such an act galvanized thousands to demand justice [12,13].

Despite the powerful presence of the concept of self-sacrifice in different cultures and Haidt's contrast of morality to self-interest, most of the recent work on moral dilemmas has largely ignored the role of the self. The most popular example is the research stemming from the trolley car problem [14]. This paradigm generally employs two versions: In the switch version a bystander can flip a switch to redirect a trolley onto another track which would otherwise kill a group of five people, but the catch is that there’s another person on the redirected track. In the footbridge version, the bystander can save the five people by pushing a single person off a footbridge in the way of the oncoming trolley. The typical finding is that people approve of action in the switch version but not in the footbridge [15–20]. Notice, however, that in both versions of the problem the life of the decision maker is not at stake. In the switch version, the decision maker is not on either of the tracks, so any choice she makes is about other people's lives. The footbridge version is somewhat more artificial, where the option for self-sacrifice of the decision maker is explicitly precluded by adding the condition that the human body which will stop the trolley needs to be very heavy, and it just so happens that there is a fat bystander nearby.

The few studies that have included the life of the decision-maker as a relevant factor in the dilemma have found somewhat mixed results. The most thorough test is in the work of Moore et al. (2008), who presented subjects with 24 dilemmas varying several different factors, one of which was self-interest. Basing their hypothesis on Petrinovich and colleagues’ [15] claim that moral intuitions often reflect evolutionary principles, the authors predicted that people would approve killing to save oneself more than killing to save others. The empirical results confirmed their prediction: when the decision-maker was a part of the group at risk, he or she was more likely to approve of sacrificing another person to save the group and the self, relative to the condition where the decision-maker’s life was not at stake. A similar role of self-interest was found by Huebner and Hauser [21] in a three-track variation of the switch version of the trolley car problem. Participants in this version were told that they could redirect the trolley toward another person, toward themselves or do nothing. A plurality of the participants chose to redirect the trolley toward the other person (48%) and away from themselves. However, there was also a substantial group (33%) that chose to redirect the trolley toward themselves. This finding was surprising for the authors of the study but to us it suggests that self-sacrifice is a vital component of the trolley car dilemma and one that needs to be addressed more carefully.

Another way to address the question of self-interest in moral dilemmas is to vary not the explicit role of the decision-maker in the dilemma, but her relationship with the people whose lives are at stake. Petrinovich et al. [15] found that people value the life of a stranger less than the life of a relative in moral dilemmas. Similarly, Swann et al. [22] found that people differentiate between in-group and out-group victims when considering altruistic self-sacrifice. That is, Spanish participants approved of self-sacrificial intervention to save the lives of other Spaniards, but less so to save the lives of Americans. Subsequent work also suggests that willingness to sacrifice one-self for in-group members is a function of identify fusion–the greater the perceived overlap between self and in-group, the greater the wiliness to sacrifice one-self [23,24].

Taken at face value, there is converging evidence that people are rather selfish in moral dilemmas. They place greater value on their own lives, the lives of relatives and the lives of in-groups than the lives of strangers. Yet, if Haidt [6, 25] is right about morality suppressing self-interest, then we should be able to find that harming others is harder than harming the self. Notice that the studies we have reviewed so far did not examine whether it is easier to push a stranger off the bridge, in the footbridge version of the trolley car dilemma, than to commit an act of self-sacrifice by jumping off yourself. Huebner and Hauser [21] directly compared sacrificing oneself versus someone else, but only in the switch version of the trolley problem. In addition they used a tri-lemma structure, which might lead to strong context effects [26] or order effects [27] In Moore et al.’s [28] experiment the decision-maker in the self-interest condition is part of a group whose lives are at risk, so not saving the self also implies not saving the rest of the group.

To the best of our knowledge, there have been no direct tests of self-sacrifice in the full version of the trolley problem. Although Huebner and Hauser [21] do report some anecdotal evidence suggesting that the option of self-sacrifice is spontaneously volunteered by several participants (e.g. “I’d jump in front, I weigh 220”) in the footbridge version of their studies, it was dismissed by the authors with the justification that “an absurd act of altruistic self-sacrifice” is suggested by participants in response to the “absurdity of the [trolley car] scenario.” This omission might easily lead to misjudging the importance of the self in moral dilemmas, or potentially inferring that people are predominately selfish when making moral choices. In this paper we provide empirical evidence from three experiments to test the degree to which people approve of self-sacrificial interventions. In Study 1, we assess approval ratings of self versus other sacrifice in both the footbridge and switch versions of the trolley car dilemma. In Studies 2 and 3, we show that sacrificing a close other may have distinct properties from sacrificing either the self or a distant other.

## Study 1

This experiment was designed to address whether people differentiate between harming the self and harming others in moral dilemmas. We used several variants of the trolley dilemma to answer this question.

### Method

#### Participants

One hundred and twenty two undergraduate students from India and the United States participated in the study for course credit. Of these, 98 students were recruited from an Indian technical university while the rest were from a large Midwestern university in the United States. The sample consisted of 96 males and the average age was 19.4 years.

#### Design and procedure

Each subject received four scenarios. The stimuli were verbal scenarios where the subjects were put in the position of an actor who has to decide whether to save several people by sacrificing the life of a single person. There were four different contexts in which the event happened, including a version of the original trolley car problem. For each context we constructed four different versions manipulating two binary factors: the type of trolley problem (switch versus footbridge) and the identity of the single victim (self versus other). This combination resulted in a total of 16 scenarios (Study 1 in S1 File). The scenarios were presented in four counterbalanced orders.

After reading each scenario participants had to indicate if they approved of the particular intervention. The answers were marked on 6-point scale where 1 was “completely disapprove” and 6 was “completely approve”. The Indian participants were asked to participate in a mass testing session, while the US participants participated in separate groups of 4 in a lab session. This study was approved by Northwestern University’s Institutional Review Board (IRB). Informed written consent were obtained under guidelines approved by Northwestern University’s IRB.

### Results and Discussion

The data were analyzed in a mixed-design ANOVA with two repeated measure factors: the type of intervention and the identity of the single victim. For the purpose of the analyses reported here, we collapsed across context as it did not systematically affect the dependent variable (for means across all contexts, please refer to the Supporting Information). Culture was treated as a between subjects factor. Missing data points were excluded case-wise.

Replicating the typical trolley effect, there was a main effect of type of intervention: intervention in the switch version was rated higher than intervention in the footbridge case (*M*_*switch*_ = 3.92; *M*_*footbridge*_ = 3.54, *F* (1, 460) = 7.57, *p* = .006, η_p_^2^ = .016). Although the self/other distinction did not have a significant main effect (*F* (1, 460) = 1.58, *p* = .21, η_p_^2^ = .003) the two factors showed a significant interaction (*F*(1,460) = 4.48, *p* = .035, η_p_^2^ = .010). Culture did not interact with any of the other variables of interest, it did have a main effect on approval ratings (F(1, 460) = 6.40, *p* = .012, η_p_^2^ = .014). American participants were more approving of intervention in general (M_Amer_ = 3.83, SD = 1.47; M_Ind_ = 3.71, SD = 1.54).

**Fig 1:**
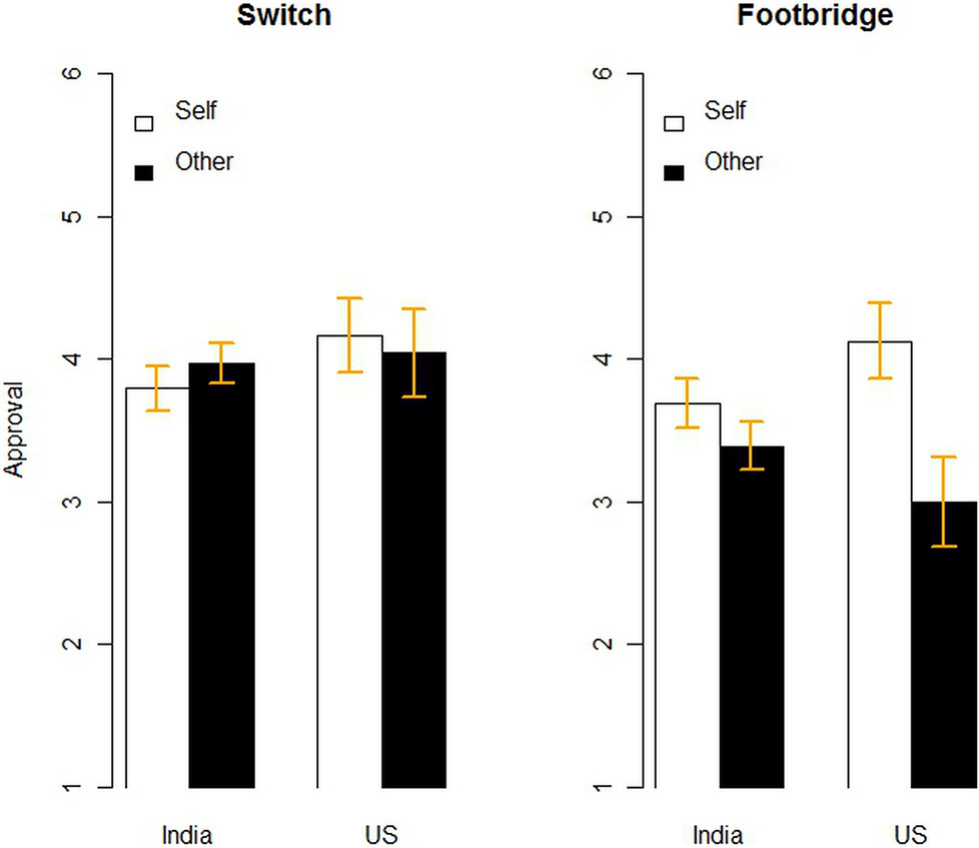
Results of Study 1. Mean approval ratings in Study 1 as a function of type of trolley car problem, self versus other sacrifice and cultural background of participants.

As shown in Fig 1, when the moral dilemma was a switch-type problem, where the harm was indirect and caused as a side effect, there was no difference between approval ratings of sacrificing the self (*M* = 3.87, SD = 1.49) versus someone else (*M* = 3.98, SD = 1.37). In the footbridge-type dilemmas, however, subjects found sacrificing the self more morally laudable (*M* = 3.78, SD = 1.58) than sacrificing someone else (*M* = 3.31, SD = 1.57).

In addition, the mean approval ratings for self-sacrifice in the footbridge version were not reliably different from overall approval in the switch version. In other words, adding the self-sacrifice option obscured the difference between the footbridge and switch versions of the trolley car problem. This study suggests that causing direct harm (as in the footbridge version) is more morally blameworthy than causing indirect harm (as in the switch version) only when that harm befalls someone else, not the self. In cases of self-sacrifice, direct harm is equivalent to indirect harm and just as worthy of praise if done to save five others.

## Study 2

The findings from Study 1 raise a number of questions, two of which we find particularly important and address in a second experiment. The first is about the scope of self-sacrifice. A broad interpretation of self-sacrifice is that it consists of inflicting cost to the self in any way possible. For instance, a mother giving up her child for some cause would be a great act of self-sacrifice because of the mental anguish or turmoil the mother would certainly experience. From this perspective, sacrificing relatives or in-groups should be similarly laudable as “pure” acts of self-sacrifice. Interestingly, several religious traditions emphasize just this sort of sacrifice, e.g. Abraham’s willingness to sacrifice Isaac, and so there is reason to accept that sacrificing a close other will result in similar judgments as sacrificing oneself.

Alternatively, one can imagine that the act of self-sacrifice is strictly limited to the boundaries of the self. In this view, an agent who harms himself (and only himself) could be seen as virtuous, but a man who sacrifices his son, his wife or his mother may be seen as a transgressor. Returning to Haidt’s [6] example, this view suggests that while sticking a pin in my hand is preferable to sticking a pin in someone else’s hand; sticking a pin in my child’s hand is worse than either of those options. In this study, we empirically test which of these perspectives is more accurate.

The second question that Study 1 raised is about the generalizability of the effect we found. Accordingly, we expanded our stimuli and used a broader set of morally relevant situations (largely based on Greene et al., [16]). We also tested Iranian subjects, expanding the population we had tested in the previous study.

### Method

#### Participants

Three hundred and sixty four students from an Iranian university and several college preparation institutions volunteered during class, and 58 students from a large Midwestern American university participated for course credit. There were 224 males in the sample and the average was 18.9 years.

#### Design and procedure

We created seven general contexts in which a single person could be sacrificed to save several others. Depending on the appropriateness of the particular situation described each context had several different variants where we manipulated the identity of the person to be sacrificed (the self, a stranger or a close relative). Each participant saw all seven of the contexts but was randomly presented with one of the variants. So, for example, for Context 1, a participant may have seen either the self-sacrifice version, the other-sacrifice version or the close relative sacrifice version. Similarly, in Context 2, the participant was again randomly presented with one of the three versions and so on and so forth for the remaining six contexts (Study 2 in S1 File). Because the assignment was random, some participants saw more self-sacrifice versions than other participants. After reading each scenario, participants judged the particular intervention on a 6 point scale, ranging from "- 3 not appropriate at all" to "3 definitely appropriate". This study was approved by Northwestern University’s IRB. Informed written consent were obtained under guidelines approved by Northwestern University’s IRB before participants started the experiment.

### Results and Discussion

The data were analyzed using a mixed design ANOVA with one within-subjects factor, the identity of the person to be sacrifice (i.e. self, other or close relative), and one between-subjects factor, participants’ cultural background (Iran or the US). Missing data were excluded case-wise.

**Fig 2:**
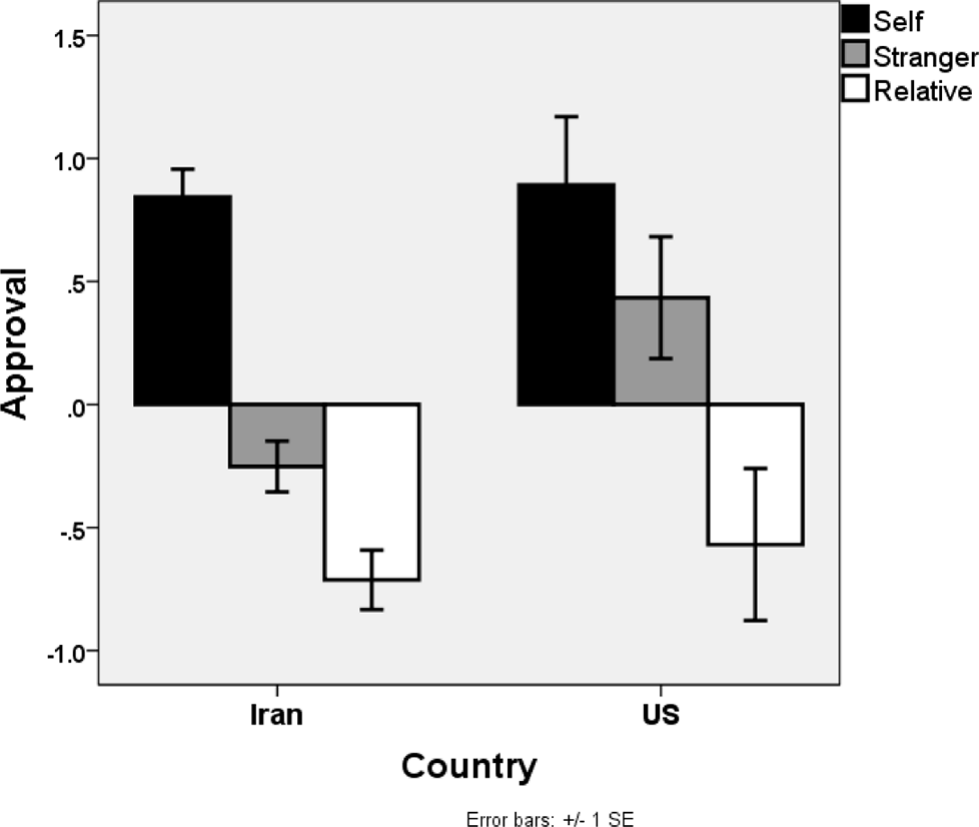
Results of Study 2. Mean approval ratings in Study 2 by person being sacrificed (e.g. self versus other versus relative) and cultural background of participants.

As predicted, we found a main effect of victim’s identity, F(2, 578) = 30.3, *p* < .001, partial η^2^ = 0.1. Collapsing across scenarios, participants approved of self-sacrifice the most (M = .82, SD = 1.78) followed by sacrificing the stranger (M = -.14, SD = 1.55) and they approved the least of sacrificing a close relative (M = -.67, SD = 1.91). As shown in Fig 2, there was no main effect of culture (F(1, 289) = 9.05, *ns*) and no interaction between culture and victim’s identity (F(2, 578) = 4.16, *ns*).

Study 2 replicates the basic patterns from the previous study while extending those results in important ways. We find that self-sacrifice is morally preferable to sacrificing a stranger however these results also suggest that sacrificing a close other is seen as more reprehensible than either of these options.

## Study 3

The results from Study 2 confirmed the findings from the first study that self-sacrifice in the trolley car problem is preferable to sacrificing someone else. This is particularly true in the footbridge version of the problem where participants prefer to jump off the bridge themselves rather than pushing off someone else. Study 2 also indicated that self-sacrifice is only morally lauded when harm is caused to the self, rather than a close other. Thus, these results begin to paint a picture that self-sacrifice is a morally praiseworthy action (compared to sacrificing others) but only when it seen as directly relevant to the boundaries of the self.

Study 3 further explores the notion of self-other asymmetry in self-sacrifice. We tested the idea that a self-sacrificial action carried out by oneself would be viewed as more moral rather than someone else. In other words, we predicted that although self-sacrifice would be favored over sacrificing someone else, the effect would be most pronounced when the action was described and seen from the 1^st^ person perspective rather than a 3^rd^ person perspective. This hypothesis extended the results of the previous two studies by examining the extent to which the moral credit of a self-sacrificial action is due to action by the self. By manipulating the visual and narrative perspective (1^st^ person versus 3^rd^ person) of a sacrificial act, we sought to answer the question–is an act of self-sacrifice committed by someone else as valued as my own?

### Method

#### Participants

One hundred eighty six participants were recruited from Amazon Mechanical Turk, an online crowd-sourcing platform. Participants were from the United States and India (n = 71 and 113, respectively, from those reporting country of residence). Females accounted for 42% of the participant pool and the average age of the participants was 32.5 years. Participants were paid 50 cents upon completion of the survey and participation lasted less than five minutes across all surveys.

#### Design and procedure

**Fig 3:**
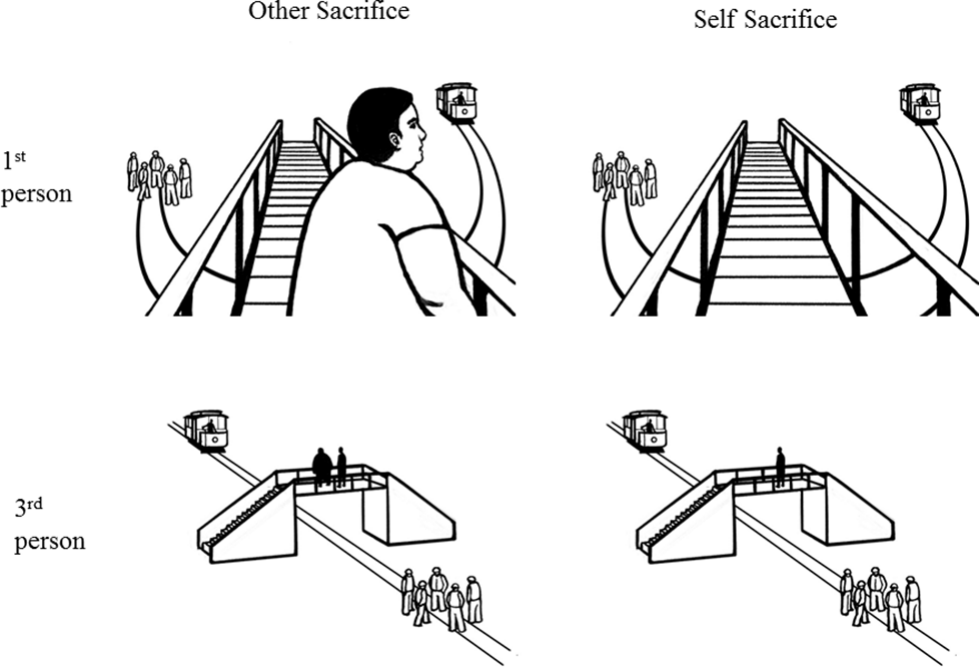
Drawings used in Study 3. Drawings used in Study 3 to manipulate visual perspective of the sacrificial action. Columns indicate the type of sacrifice depicted in the pictures while rows show the perspective manipulated.

Participants were randomly assigned to one of four between-subject conditions in which we manipulated the Type of Sacrifice (Self versus Other) and Perspective (1^st^ person versus 3^rd^ person). All scenarios were variants of the footbridge version of the trolley car problem. Each participant received a pictorial representation of the situation and a brief verbal description. The pictures used in this study are shown in Fig 3 (please refer to Study 3 in S1 File for the full text of the scenarios). In each scenario, the actor was said to have engaged in either the self or other-sacrificial action. After reading the scenarios and referring to the pictures, participants were asked to rate how much they approved of the action on a scale of 1 to 6 with higher numbers indicating greater approval. This study was approved by Northwestern University’s IRB in addition to Iranian Institute for Cognitive Science Studies IRB. Informed written consents were obtained before the experiment under guidelines approved by Northwestern University’s IRB in addition to Iranian Institute for Cognitive Science Studies IRB.

### Results and Discussion

**Fig 4:**
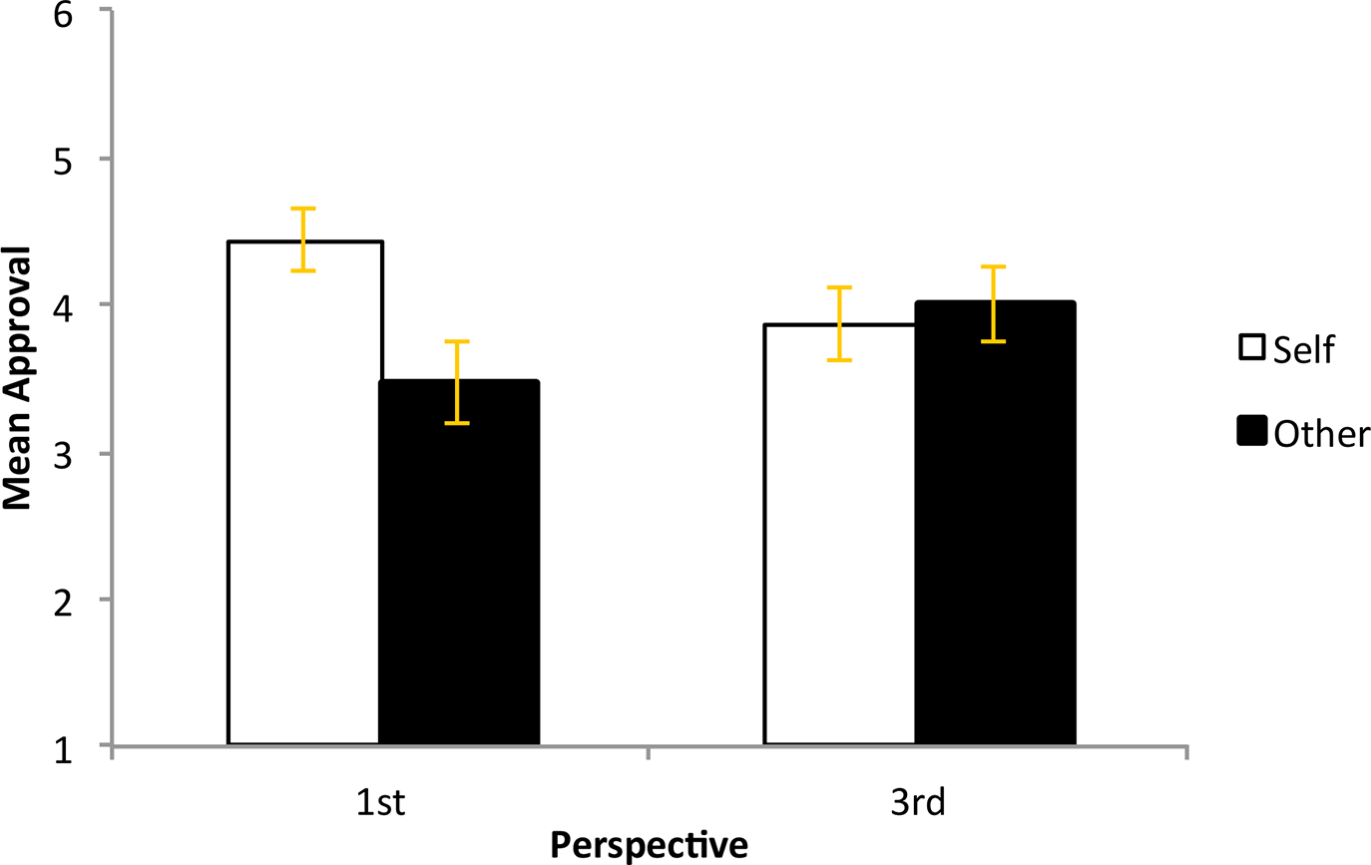
Results of Study 3. Mean approval ratings in Study 3 by type of sacrifice (self versus other) and perspective (1^st^ person versus 3^rd^ person).

Two participants were excluded from the analyses because of missing data. We analyzed the data using a three-factor, between-subjects ANOVA with country of residence, type of sacrifice and perspective as fixed factors. The predicted interaction between the Type of Sacrifice (Self versus Other) and Perspective (1^st^ person versus 3^rd^ person) was observed (F(1, 176) = 5.28, *p* = ..023, η_p_^2^ = .029). Participants preferred self-sacrifice to other-sacrifice but only when it was shown and described from a first person perspective (M = 4.44 versus 3.47, SD = 1.57 and 1.87, respectively). None of the other main effects or interactions were statistically significant (please refer to Fig 4). Study 3 provides further evidence that self-sacrifice in moral dilemmas is viewed as a praiseworthy action compared to sacrificing someone else. However, self-sacrifice is valued only within the specific boundaries of the self, the further away one moves from this boundary as in the third person perspective in this study, the less significant self-sacrifice appears to be. As noted by a helpful reviewer, the shift in psychological distance by manipulating visual and narrative perspective [29, 30] may remove the emotional salience of the footbridge dilemma, making the third-person scenario akin to the switch version. Without the visceral engagement of the first person scenario, the harm may not be considered as severe and the self-sacrifice not as admirable. Thus, the results of Study 3 are rather analogous to the comparison made in Study 1, between the footbridge and switch versions of the trolley, even as the present study aimed to target perspective.

## General Discussion

The results from the studies presented here have three main implications. The first one is an empirical demonstration that in moral dilemmas involving direct harm, sacrificing another person is considered less appropriate than sacrificing the self. This result was observed across three experiments and among different cultural groups. Our findings suggest that Moore et al. [28] and Huebner and Hauser’s [21] results that people are rather selfish in moral dilemmas are not broadly generalizable. In the footbridge scenario, people rate jumping off the bridge to save five others as more morally appropriate than pushing someone. In plain numbers, the calculus of five for one may appear the same, but the difference between self-sacrifice and murder appears to be an important one for our participants. Because we used a somewhat different methodology, our results do not necessarily disagree with the particular findings of previous studies on self-interest in moral dilemmas. Unlike Moore et al. [28], we did not control self-interest by stating that the decision maker was part of a group, confounding possible in-group loyalty with self-interest. Instead, we compared cases where the decision maker had to sacrifice another person, or sacrifice himself to save another group of people, and found that in footbridge cases sacrificing another person was approved less than self-sacrifice. While such results are novel, and somewhat surprising given previous findings, they can be predicted by Haidt's definition of morality, as the opposite of self-interest.

In addition to providing an empirical challenge of previous claims about self-interest in moral dilemmas, these results have implications for current theories on moral decision making. Most of the recent work in moral psychology stems from the presupposition that normative principles are agent-neutral [31,21]. According to the Golden Rule [32], for example, which is arguably the basis of our current understanding of universal human rights, one should treat others as she wishes others to treat her–essentially minimizing the self-other distinction. Yet, we found that this distinction is non-trivial, and in cases of direct harm, sacrificing the self is different from sacrificing another. While previous work has already demonstrated that judgments might depend on the particular role of the moral agent [33] and that the moral obligations of one person are not necessarily seen as obligations for another [34], the self-other distinction in our results further emphasizes the role of agent-relativity of moral choices. In other words, all other things being equal, I might choose to sacrifice myself, but I might disapprove of sacrificing someone else, or someone else sacrificing me.

Third, we found that the preference for self-sacrifice may not necessarily be a preference for inflicting high self-cost [35]. If self-sacrifice is just an extreme form of costly signaling, then we could expect that both sacrificing the self and sacrificing a relative would be approved more than sacrificing a stranger. Yet, in Study 2 we found that sacrificing a stranger is harder than sacrificing the self, but easier than sacrificing a relative. The self-relative-other pattern highlights the role of agent-relativity in moral judgments, suggesting that both the role of the self as agent, and the relationship of the self to the patients of the action matter. Future analysis of the causal component of moral judgments [36,37] should take into account the unique causal role that the self can have in moral dilemmas. Including the life of decision maker in causal structure and manipulating his relationship to the other parties concerned presents a challenge for some of the main causal distinctions used to analyze the structure of a moral dilemma, such as victim and harm, agent, and patient, or means and ends.

Before we conclude, we need to address two important caveats. The first one is related to the experimentally controlled, but admittedly artificial nature of the scenarios involved. Our data suggest that the self is an important factor for moral judgments about hypothetical events, but future work is needed to show how well such finding will generalize to real life situations (see 9). As others have pointed out, while the relatively narrow field of “trolleyology” has uncovered important parameters underlying moral cognition, it relies almost exclusively on hypothetical scenarios and abstract moral judgments, rather than actual moral behavior. The present studies are subject to similar criticism. Participants in these studies were asked about the moral appropriateness of various hypothetical scenarios which may differ from their moral decisions or behaviors in real world situations. In particular, Tassy et al. [38] show that action choices, in moral dilemmas like the trolley car problem, often result in more utilitarian decisions than judgment choices. This indicates that if participants were to encounter the situations that we have employed in the current set of studies, they might be more willing to endorse self-sacrifice particularly if the alternative was to sacrifice someone else. This is an intriguing, albeit untestable, hypothesis yet, as with other trolley-based moral scenarios, we hope that the inclusion of self-sacrifice in these scenarios reveals an important distinction which has thus far been under-represented in the literature.

The second caveat is related to the possibility for strong context effects. There may be very specific norms guiding the balance between self-sacrifice and self-interest. Jumping on a grenade to save five fellow soldiers can easily be seen as praiseworthy act, yet a healthy soldier donating all his body organs to save the other soldiers will probably be seen not as a hero but as an aberration. Varying the structure of the dilemma in which self-sacrifice occurs, for instance by using variants of the Volunteers Dilemma, might also yield further insights into the contexts in which self-sacrifice is morally laudable and when it perceived as foolish or worse, an immoral action.

## Conclusion

Part of the challenge for moral psychologists is to choose which factors to study. Deciding if self-sacrifice and self-interest are important for moral research is largely a question of definition and theoretical framework, yet we have shown that including the self as both agent and victim virtually eliminates the trolley car effect. We also found that it is not simply due to self-cost, because sacrificing a relative seemed to be very different from sacrificing the self. Additionally we found that the incongruent visual perspective of a third person self-sacrifice eliminated the positive value of a self-sacrificial action. These results have important theoretical implications for the field of moral psychology, prompting future work on agent-relativity of moral judgments, and a more thorough analysis of the role that causality plays. In addition to theoretical importance, the distinction between self and other in the moral domain might have relevance for real world behaviors in their extreme forms, ranging from heroic altruistic acts [39] to self-sacrificial terrorism [40,41].

## Supporting Information

S1 File Scenarios for the three experiments. (PDF)

S2 File Data for the three experiments. (XLS)
