# Supplementary material for: The Role of Self-Sacrifice in Moral Dilemmas
Source: PLoS One. 2015 Jun 15;10(6):e0127409. doi: 10.1371/journal.pone.0127409 (PMC4468073; doi:10.1371/journal.pone.0127409)
Supplement: S1 File — (PDF) [file pone.0127409.s001.pdf]

Supplementary Materials: Study 1

There were 4 different content types (trolley, zoo, firefighter and soldier) and 4 experimental conditions (footbridge vs. switch, and self vs. other). Each participant received 4 scenarios, one of each content type and one of each experimental condition. One variant of each content type is shown below.

Trolley/Footbridge/Other
A runaway trolley is heading down the tracks toward five workmen who will be killed if the trolley proceeds on its present course. You are on a footbridge over the tracks, in between the approaching trolley and the five workmen. Next to you on this footbridge is a stranger who happens to be very large, much larger than you are.
The only way to save the lives of the five workmen is to push this stranger off the bridge and onto the tracks below where his large body will stop the trolley. The stranger will die if you do this, but the five workmen will be saved.

Firefighter/ Footbridge/Self
You are a firefighter who is rescuing people in a tall building after a devastating earthquake. Four people are trapped on the remnants of a wooden deck and cannot get to safety since one of the deck's ends has no support and if they move the whole deck will fall down. You can jump on the deck and use your own weight to counterbalance the 4 people who will be able to escape. If you do this, however, after the 4 leave the deck will fall down and you will die.

Zoo / Switch / Other
You are an animal keeper in a zoo when an accident happens. The elephant runs free and goes on a killing spree. It is heading toward 6 people who are hiding in a wooden cabin and will inevitably be killed. You can save the 6 by opening a gate so the elephant will run in an adjacent compartment, where it will kill one person.

Soldier/Switch/Self
You are a scout on the enemy territory which observes the evacuation of five fellow soldiers. They are being chased by a larger enemy unit and will soon be encircled and destroyed. The only way to help them is if you call for an air strike, which will delay the enemy unit, but will also kill you.

Supplementary Materials: Study 2

We created 7 different contexts, where a single person is sacrificed to save someone else's life. In addition, each context had between 2 and 6 versions, depending on who is the person being sacrificed. In some versions this person is the self, in others a relative, or yet in others, a stranger. Each participant saw one scenario of each of the seven contexts, randomly assigned to the particular version of that scenario. The table below shows the mean approvals for each of the versions.

Table SM1. Mean approval and standard deviations for each scenario by culture. Negative numbers mean stronger disapproval.

			Iran		USA	
Context	Sacrifice	Code	mean	S.D.	mean	S.D.
crying baby	relative	1_r_n_b	0.02	2.24	-0.36	2.25
crying baby	other	1_o_d_m	-0.79	2.24	0.43	1.81
crying baby	other	1_o_n_m	-0.90	2.31	1.38	1.41
crying baby	realtive	1_r1_n_b	-0.47	2.04	1.00	1.76
crying baby	other	1_o1_d_m	-0.63	2.28	2.00	2.45
crying baby	other	1_o1_n_m	-0.58	2.25	0.50	1.93
injured soldier	other	2_o_n_b	-0.08	2.29	-0.10	2.56
injured soldier	other	2_o_d_m	-0.25	2.32	0.91	2.02
injured soldier	relative	2_r_n_m	-1.16	2.11	1.11	1.69
injured soldier	self	2_s_n_m	-0.05	2.34	0.73	2.15
injured soldier	other	2_o1_n_m	-1.22	2.23	0.30	2.58
injured soldier	relative	2_s1_n_m	0.81	2.27	1.00	2.52
footbridge trolley	other	3_o_n_b	-1.41	2.03	-0.54	2.23
footbridge trolley	other	3_o_d_m	-1.36	2.03	-1.09	2.20
footbridge trolley	self	3_s_n_m	0.36	2.02	-0.17	1.99
Sophie's choice	relative	4_r_n_b	-1.07	2.18	-1.56	1.88
Sophie's choice	other	4_o_n_m	0.39	2.35	0.37	2.24
Sophie's choice	other	4_o_d_m	-1.02	2.29	-1.20	1.48
Sophie's choice	relative	4_ro_d_m	-0.86	2.21	-0.60	2.07
Sophie's choice	relative	4_ro1_d_m	-0.64	2.17	0.00	2.31
switch trolley	other	5_o_n_b	1.34	1.90	1.57	1.59
switch trolley	self	5_s_n_m	1.05	2.03	1.28	1.71
switch trolley	other	5_o_d_m	1.54	1.70	0.75	1.91
army doctor	other	6_o_n_m	-0.50	2.20	-0.90	1.97
army doctor	self	6_s_n_m	0.73	1.98	0.33	2.18
army doctor	self	6_s1_n_m	1.14	1.84	1.26	1.76
army doctor	other	6_o1_n_m	0.95	1.99	0.06	2.29
king's servant	relative	7_r_n_m	-0.69	2.19	-1.00	1.96
king's servant	other	7_o_n_m	-0.16	2.24	-0.03	1.88

Supplementary Materials: Study 3

Participants received a set of instructions before viewing the scenario and associated visual manipulation.

Other Sacrifice – First Person Perspective: A runaway trolley is heading down the tracks toward five workmen who will be killed if the trolley proceeds on its present course. You are on a footbridge over the tracks, in between the approaching trolley and the five workmen. Next to you on this footbridge is a stranger who happens to be very large, much larger than you are. The only way to save the lives of the five workmen is to push this stranger off the bridge and onto the tracks below where his large body will stop the trolley. The stranger will die if you do this, but the five workmen will be saved. Is it appropriate for you to push the stranger on to the tracks in order to save the five workmen?

Other Sacrifice – Third Person Perspective: A runaway trolley is heading down the tracks toward five workmen who will be killed if the trolley proceeds on its present course. A man is standing on a footbridge over the tracks, in between the approaching trolley and the five workmen. Next to him on this footbridge is a stranger who happens to be very large, much larger than he is. The only way to save the lives of the five workmen is for the man to push this stranger off the bridge and onto the tracks below where his large body will stop the trolley. The stranger will die if the man does this, but the five workmen will be saved. Is it appropriate for the man to push the stranger on to the tracks in order to save the five workmen?

Self Sacrifice – First Person Perspective: A runaway trolley is heading down the tracks toward five workmen who will be killed if the trolley proceeds on its present course. You are on a footbridge over the tracks, in between the approaching trolley and the five workmen. The only way to save the lives of the five workmen is to jump off the bridge and onto the tracks below where your body will stop the trolley. You will die if you do this, but the five workmen will be saved. Is it appropriate for you to jump on to the tracks in order to save the five workmen?

Self Sacrifice – Third Person Perspective: A runaway trolley is heading down the tracks toward five workmen who will be killed if the trolley proceeds on its present course. A man is standing on a footbridge over the tracks, in between the approaching trolley and the five workmen. The only way to save the lives of the five workmen is for this man to jump off the bridge and onto the tracks below where his body will stop the trolley. He will die if he does this, but the five workmen will be saved. Is it appropriate for the man to jump on to the tracks in order to save the five workmen?
